# Patient experiences with interventions to reduce surgery cancellations: a qualitative study

**DOI:** 10.1186/1471-2482-13-30

**Published:** 2013-08-08

**Authors:** Einar Hovlid, Christian von Plessen, Kjell Haug, Aslak Bjarne Aslaksen, Oddbjørn Bukve

**Affiliations:** 1 Institute of Social Science, Sogn og Fjordane University College, Postbox 1336851 Sogndal, Norway; 2 Department of Public Health and Primary Health Care, University of Bergen, Bergen, Norway; 3 Department of Thoracic Medicine & Infectious Disease, Hillerød Hospital, Hillerød, Denmark; 4 Department of Health Studies, Faculty of Social Sciences, University of Stavanger, Stavanger, Norway; 5 Department of Radiology, Haukeland University Hospital, Bergen, Norway; 6 Institute of Surgical Sciences, University of Bergen, Bergen, Norway

**Keywords:** Quality improvement, Surgery, Cancellations, Patient centered, Qualitative

## Abstract

**Background:**

The cancellation of planned surgery harms patients, increases waiting times and wastes scarce health resources. Previous studies have evaluated interventions to reduce cancellations from medical and management perspectives; these have focused on cost, length of stay, improved efficiency, and reduced post-operative complications. In our case a hospital had experienced high cancellation rates and therefore redesigned their pathway for elective surgery to reduce cancelations. We studied how patients experienced interventions to reduce cancellations.

**Methods:**

We conducted a comparative, qualitative case study by interviewing 8 patients who had experienced the redesigned pathway, and 8 patients who had experienced the original pathway. We performed a content analysis of the interviews using a theory-based coding scheme. Through a process of coding and condensing, we identified themes of patient experience.

**Results:**

We identified three common themes summarizing patients’ positive experiences with the effects of the interventions: the importance of being involved in scheduling time for surgery, individualized preparation before the hospital admission, and relationships with few clinicians during their hospital stay.

**Conclusions:**

Patients appreciated the effects of interventions to reduce cancellations, because they increased their autonomy. Unanticipated consequences were that the telephone reminder created a personalized dialogue and centralization of surgical preparation and discharge processes improved continuity of care. Thus apart from improving surgical logistics, the pathway became more patient-centered.

## Background

The cancellation of planned surgeries is a well-recognized quality problem. High cancellation rates may indicate that scarce health resources are being used ineffectively, thereby increasing costs [1,2]. Patients are directly affected by cancellations; they increase waiting times and may lead to harmful delays of operations [2,3]. Further, the cancellation and the extra waiting time may cause physical and emotional distress [4].

Previous research has addressed how cancellations can be reduced through earlier and better clinical pre-assessment and improved surgical scheduling [5-9]. Interventions to reduce cancellations have been evaluated from a management and a medical perspective focusing on cost, length of stay, improved efficiency, and reduced post-operative complications [6,7,9-12]. To our knowledge, the effects of interventions to reduce cancellations have not been explored from the perspective of those who are affected by them - the patients.

We have previously reported the case of a Norwegian district general hospital that redesigned its pathway for elective surgery and achieved a sustained reduction in cancellations [13,14]. The purpose of the current study was to explore patient experiences with the interventions to reduce the cancellations.

## Method

### Theoretical basis for interventions to reduce cancellations

Reducing cancellations is a complex task because the causes are multifactorial, i.e. they are related to patients, organizational issues and clinical staff [1,15]. Common causes for cancellations are related to patients’ medical conditions, inadequate medical pre-assessment, overbooking of lists, facility shortcomings, and patient non-attendance [1,6,16]. Because the reasons for cancellations are multi-factorial, interventions to reduce cancellations need to take into account the complexity of the problem. The literature suggests that most cancellations can be avoided by redesigning work processes, improving planning and coordination, and performing earlier clinical pre-assessment of patients [16,17]. It has also been suggested that patients themselves should select the date of surgery, receive early notice of their operating day, and a reminder of their appointment [17]. Involving patients in these ways may even increase their satisfaction with treatment decisions during initial consultations, which is a strong predictor of attendance for surgery [18].

### Design

We did not find relevant literature about patient experiences with interventions to reduce cancellations. Thus, we chose a qualitative design with semi-structured interviews to explore the field [19]. Moreover qualitative methods are useful in evaluative studies because they are open to unexpected inputs [20].

We conducted a comparative case study and interviewed patients from two hospitals, A and B [21]. In hospital A the pathway for elective surgery was redesigned and in hospital B it remained unchanged. This design enabled us to get rich data about patient experiences. Furthermore, we could isolate the effects of the interventions and establish a probable relationship between the interventions and patient experiences.

### Description of the case

Hospital A is a district general hospital where the pathway for elective surgery was redesigned. It has seven operating suites and 34 surgical beds. Hospital B, is a local hospital with three operating suites and 14 surgical beds. The two hospitals belong to the same local health authority and have the same senior management team. Initially, both hospitals had a similar clinical pathway for elective surgery and faced the same quality problems with their services. As a consequence, the health authority planned a redesign of the pathways at both hospitals. For practical reasons, the revised plan was abandoned at hospital B.

Hospital A redesigned its pathway through the following interventions: earlier clinical pre-assessment, improved flow of information among surgeons and anesthesia personnel, patient participation in selecting the date for surgery, centralization of preparation and discharge of patients to a single unit, a telephone call to patients two days prior to surgery, and a common computer-based system for scheduling operations across all surgical departments [13]. The mean cancellation rates at hospital A and B after the interventions was respectively 4,9% and 6,1%. Table 1 displays the main differences between the original and the redesigned pathways.

**Table 1:** **Main differences of the pathway for elective surgery before and after redesign (based on Table**2**in Hovlid et al. 2012**[13]**)**

**Time periode**	**Clinical pathway before redesign**	**Clinical pathway after redesign**
Consultation at outpatient clinic	Medical pre-assessment done the day before surgery.	Surgeons and anesthesia personnel did conjointly establish a new routine that clarified the responsibilities and division of labor between them.
	Patients cleared for surgery were sent home without an appointment for surgery and without a medical pre-assessment.	Patients participated in planning the date of surgery and obtained the actual appointment while at the outpatient clinic.
Consultation at drop-in anesthesia outpatient clinic at day-surgery center	Not applicable	A new day-surgery center established within existing premises.
		Patients cleared for surgery proceeded directly to the laboratory for blood tests and medical pre-assessment at newly established drop-in anesthesia outpatient clinic at the day-surgery center.
		Surgeon’s dictated notes written immediately after consultation for anesthesia personnel to have information at hand during preoperative assessment.
Waiting for surgery	A letter with appointment for surgery was sent to the patient. Patients had no influence on appointment time.	Patients received a phone call from the hospital two days prior to surgery to ensure they were fit and ready.
	Limited planning across different surgical departments. Each department had their own surgery program that was not accessible on-line	One common electronic surgery planning system and the position of a coordinator for all surgical departments established.
Surgery	Patient showed up for pre-assessment the same day or one day in advance of the planned surgery.	All patients scheduled for elective surgery received at day-surgery center.
	Routines varied across departments.	Pre-surgery preparations standardized.
After surgery	Variation of discharge process in different department.	All day-surgery patients were discharged from the day-surgery center through new standardized routines.
	Discharge letter not always ready when the patient left.	Discharge letter written and given to the patient before discharge.

### Recruitment

Clinicians at hospitals A and B recruited patients for the study. They handed out an information letter describing the purpose of the study during the pre-surgical medical assessment. Patients who agreed to participate signed an informed consent form. The clinicians returned the form by mail and the first author called the patients after they had completed their surgery. We recruited 10 patients at hospital A and eight patients at hospital B.

### Data collection

Between January and March 2011, the first author conducted semi-structured telephone interviews with patients who had undergone operations at hospitals A and B. For patients under 18 years of age, the first author interviewed a parent. We purposively sampled patients with different characteristics with regard to gender, age and type of surgery (day surgery/in-patient-stay) [22]. The interviews took place 1 to 7 months after the patients had completed their surgery.

The interviews followed a guide with open-ended questions to explore the experiences of the patients. The guide was based on a literature review about patient experiences of interventions to improve care [23-26], the interventions implemented to reduce cancellations at hospital A and on the different phases of elective surgery, the consultation at the out-patient clinic, the time spent waiting for surgery, and the hospital admission for surgery. The guide is enclosed in the Additional file 1.

The first author made consecutive case notes of the interviews. Furthermore, we started analyzing the data during the data collection. Thus the data collection and data analysis were iterative steps. From the case notes and our analysis, we observed that the last two interviews did not add any new information, i.e. we had reached saturation of our data. We then concluded that the sample size was sufficient for the purpose of this study and stopped recruiting patients [19,27,28].

### Analysis

We audio-taped the interviews, transcribed them verbatim, and transferred them to HyperRESEARCH 2.8.3 computer software (ResearchWare, Inc., 2009) for coding. We performed a content analysis using a direct approach, as described by Hsieh and Shannon [29]. Based on the theory about interventions to reduce cancellations, we developed a coding scheme to reflect the interventions implemented at hospital A, i.e. earlier clinical pre-assessment, patient participation in scheduling the surgery, telephone calls to patients prior to surgery, and centralized preparation and discharge. The first author coded the interviews and identified passages where the patients described experiences related to these interventions.

The last author read all the interviews and validated the coding; the first and last authors then compared codes from the two hospitals. The aim was to identify how the patients’ experiences were related to the interventions that reduced cancellations at hospital A. Using an iterative process of coding, then reflecting on the codes and condensing, the first and last authors identified common themes relating to how the patients had experienced these interventions [30].

A professional bilingual translator translated the quotations in this article from Norwegian into English. Quotations were adapted from an oral style to a written format to enhance readability without changing the content or meaning [31].

### Ethical considerations

Patients participated after informed, written consent and could withdraw from the study at any time. The Western Department of the Regional Committee for Medical and Health Research Ethics in Norway deemed a full ethical review unnecessary because sensitive patient data were not included in the study. The study protocol was accepted by the Norwegian Social Science Data Services, which reviewed ethical aspects relating to the collection and handling of data (e.g. voluntary participation based on informed consent, anonymity of informants, and appropriate storage of data).

## Results

We completed interviews with 16 of the 18 patients (12 patients, 2 mothers and 2 fathers), eight from hospital A and eight from hospital B. One patient withdrew his/her consent to participate and one interview was not completed because of technical difficulties. Table 2 displays the characteristics of the interviewees.

**Table 2:** Interviewee characteristics

**Characteristic**	**Value**	**Number of interviewed patients at hospital A (redesigned pathway)**	**Number of patients interviewed at hospital B (original pathway)**
Age (year)	<18	4	0
	18–39	2	0
	40–59	2	4
	60+	0	4
Sex	Men	5	4
	Women	3	4
Type of surgery	Day surgery	4	5
	Hospitalized	4	3

We identified three common themes concerning how patients experienced the interventions. These included 1) the importance of being involved in scheduling time for surgery; 2) individualized preparation before the hospital admission; 3) the importance of establishing relationships with a minimum number of clinicians during the hospital stay. We have structured the presentation of our findings around these themes and present patient quotes to illustrate how they experienced the changes. Additional quotes illustrating our findings can be found in the Additional file 2.

### The importance of being involved in scheduling time for surgery

Patients at hospital A originally received their surgical appointments by mail, after the consultation at the out-patient clinic when the decision to operate was made; so they could not participate in its planning. This process was changed so that patients could choose the date of surgery and confirm the appointment during the actual pre-operative consultation. The patients reported that this option was important to them, because the elective surgery had an impact on them and their social surroundings beyond the medical condition and its treatment. The surgery affected the way each patient planned and lived their lives. The active participation in deciding the date of surgery, in combination with agreeing on the appointment in advance, allowed them to make choices to fit their personal circumstances. It allowed them to integrate the planned surgery into their lives. Patients from hospital B who were not given this opportunity, expressed that they would have preferred to participate in scheduling their surgery, and pointed to the importance of knowing the actual date of surgery earlier on.

The following quotations illustrate this finding:

Interviewer (I): Did you have any influence over the scheduling of your surgery?

Patient (P) at hospital A: Yes, I did. And that was really good. I was due for a training period, and was able to work around that. I couldn’t have made it work otherwise.

I: Did you have any influence over the scheduling of your surgery?

P at hospital B: No, but that would have been a great practical advantage, as it would have made it possible to schedule around work, school, and traveling to the hospital. It is important to be able to plan ahead.

### Individualized preparation before hospital admission

At hospital B, patients received practical information about their forthcoming operation in the same letter that announced the date of their operation. The patients at hospital B reported that they were satisfied with the information they received prior to surgery. Patients at hospital A received the same type of information at their out-patient consultation when the decision to perform surgery was made. In addition, hospital A started calling patients two days prior to surgery to make sure they were in good health and would keep their appointment. If patients were unable to attend their scheduled surgery, another patient was rescheduled, thereby avoiding a cancellation.

The telephone calls also had beneficial effects beyond preventing cancellations. They created a dialogue between the patient and the hospital. Patients could ask questions, staff were able to support their pre-operative preparation, and check if the patient had understood the information. Patients felt that the telephone call demonstrated that the hospital cared about their well-being and was prepared and ready for their particular situation.

The following quotation illustrates these experiences:

P (Hospital A): It was a very positive thing. I felt that somebody cared about what was going to happen and that they were more on top of things than if they had just sent a letter.

### Relating to fewer clinicians

Hospital A established a surgical center to reduce cancellations arising from poor planning and a lack of coordination between departments. Preparation and discharges of all patients were centralized to this facility to optimize resource utilization and to ensure improved planning and coordination. Through the centralization patients had to relate to fewer health professionals during their admission, because they no longer were admitted to a regular ward. Patients from both hospitals emphasized the importance of having relationships with a limited number of health professionals, because it contributed to continuity of care and made them feel safe.

P at hospital B: One thing I thought was really good, was that the same people were there when you came in and when you woke up again. I have had a number of surgeries over the years. On other occasions, I felt like I constantly had to relate to new people, and that was downright pathetic.

I: Why is meeting the same people the whole time a good thing?

P: I think it gives a sense of security. A person met you, knows about you, and follows your progress.

## Discussion

Our main finding was that patients appreciated the effects of interventions that had been implemented to reduce cancellations. Interestingly, patients also described positive effects that the improvement team had not planned for, such as improved continuity of care and the personalized dialogue prior to surgery.

Patients appreciated the changes because they contributed to making care more patient-centered. Patient-centeredness is a core value of health care and one of the main characteristics of high quality health services [32]. It has been defined as “ . . . respecting and responding to patients’, wants, needs and preferences, so that patients can make choices in their care that best fit their individual circumstances” [32,33]. Patient-centeredness is important in its own respect, but has also been associated with better clinical outcomes [34]. In line with previous research, we found that patient participation in scheduling surgery and timely scheduling was important to them [35,36]. This change responded to patients’ wants, needs, and preferences and enabled them to make and adapt choices that fitted their individual circumstances, thus making the service more patient-centered.

Hospital A (redesigned pathway) established telephone calls prior to surgery to reduce non-attendance, in line with published research about telephone reminders [37]. For patients, the telephone calls had additional effects in that they changed the information flow from a one way communication style (letter) to a personalized dialogue. Another unforeseen effect was related to the centralization to the surgical center at hospital A, which was implemented to improve coordination and efficiency. For the patients this change reduced the number of professionals they had to interact with.

Health care needs to reduce costs and improving efficiency, while maintaining or improving quality [38]. This challenge has stimulated an interest in how quality improvement may increase efficiency [39,40]. Moreover modern health care needs to deliver care that is more patient-centered [32,41]. Improved efficiency and patient-centeredness are sometimes considered contradictory, because interventions focusing solely on cost and efficiency can affe ct care in ways that health professionals and patients do not approve [42]. At hospital A (redesigned pathway), cancellations were reduced and the number of operations per month increased, while resources remained unchanged, which indicates that efficiency has increased [13]. The interviewees in our study told us that they appreciated the service at hospital A. So it seems that interventions to reduce cancellations can be designed in ways that not only improve the logistics of surgical planning and efficiency, but also can make care more patient-centered.

### Limitations and further research

We are unable to describe any potential negative effects from the interventions to reduce cancellations, because all the patients gave only positive feedback. We asked open-ended questions about their general experiences and covered all of the aspects within the trajectory of elective surgery. Also, our interview guide was based on theory about reducing cancellations and patient experiences to improve overall care. Thus the interviewees certainly had the opportunity to mention negative experiences and we assume that our results are valid in spite of the absence of negative feedback.

The clinicians recruited the patients for the study at the outpatient clinic when the decision to perform surgery was made. All these patients proceeded to surgery Thus we do not have information on the experiences of patients who had their surgery cancelled. This is obviously a limitation of our study because such patients could have had different and diverging experiences.

Interviewees at the hospital with the new clinical pathway were generally younger than at the hospital with the original pathway. This imbalance in age distribution could have affected our data because experiences can vary by age group. The patients at the hospital with the original pathway did, however, ask for the same kind of changes, which were made at hospital A.

Another limitation of our study was that our interview guide was based solely on theory. This could restrain our data collection. Methods to avoid this could have been to use inputs from patients, e.g. a focus group when developing the guide. Meanwhile, the long travel distances in rural Norway, did not allow us to gather patients for a focus group before finalizing the questions in the interview guide. We tried to compensate for this limitation by asking open ended questions and included a question at the end where the patients could tell about any experiences they considered important. Thus, we conclude that we collected valid data to answer our research question.

Interviews over the telephone may yield less information than face to face encounters, because researchers have limited insight into and influence on, the interviewees’ circumstances and reactions [43]. Face-to-face interviews were not feasible because of the long distances to travel in rural Norway. Also, we did not want to interview patients during the psychologically vulnerable phase immediately before or after their operation. The first author, who conducted all of the interviews, has extensive experience in communicating over the telephone with patients in the clinical setting, and has conducted previous studies using telephone interviews. Thus, in spite of the inherent limitations of telephone interviews, we are confident that we collected valid data to answer our study questions.

Saturation of data can be observed prematurely if a sample is not sufficiently diverse [27]. We interviewed patients of both genders, with different ages, and a range of operations types. Thus we conclude that our sample was diverse enough to capture a wide range of experiences [22].

Findings from this exploratory study need to be validated in larger studies. Such research could benefit from a prospective design and by using mixed methods. Patient participation in decisions about appointments and pre-admission telephone calls should have relevance for ambulatory care in general; particularly in health care systems where patients cannot freely choose where to be treated. Future studies should address patient experiences with these interventions in other settings. Further, patients who have valuable first-hand ‘expertise’ are not often directly involved in planning changes. More research is needed about how to effectively involve patients in developing patient-centered care models [24,44].

## Conclusions

Our findings indicate that patients appreciated the effects of interventions to reduce cancellations, because they increased their autonomy by enabling choices that fitted with their particular circumstances. The interventions also had unanticipated consequences; the telephone reminder created a personalized dialogue prior to surgery and centralization of surgical preparation and discharge processes contributed to continuity of care. Thus apart from improving surgical logistics, the interventions to reduce cancellations contributed to increased patient-centeredness of the care.

## Competing interests

The authors declare that they have no competing interests.

## Authors’ contributions

EH designed the study, collected the data, analyzed the data and drafted the manuscript. OB participated in the design of the study, the data analysis and helped to draft the manuscript. KH participated in the design of the study and helped to draft the manuscript. ABA participated in the design of the study and helped to draft the manuscript. CVP participated in the design of the study and helped to draft the manuscript. All authors read and approved the final manuscript.

## Pre-publication history

The pre-publication history for this paper can be accessed here:

http://www.biomedcentral.com/1471-2482/13/30/prepub

## Supplementary Material

Additional file 1 Interview guide. Click here for file

Additional file 2 Additional quotes from patients. Click here for file
