# Supplementary material for: Patient experiences with interventions to reduce surgery cancellations: a qualitative study
Source: BMC Surg. 2013 Aug 8;13:30. doi: 10.1186/1471-2482-13-30 (PMC3750692; doi:10.1186/1471-2482-13-30)
Supplement: Additional file 1 — Interview guide. [file 1471-2482-13-30-S1.docx]

**Interview guide - patient experiences with interventions to reduce surgery cancellations**

**Background information which was collected:**

Gender, age, day surgery/hospitalized, date for surgery, previous experience with surgery

**Before consultation at outpatient clinic**

How did you experience the information from the hospital prior to your consultation at the outpatient clinic?

**Consultation at outpatient clinic**

How did you experience the consultation at the outpatient clinic? (the consultation when the decision to perform surgery was made)

How were you as a patient involved during the consultation?

What was your experience regarding deciding the time for the operation?

What kind of information did you receive during the consultation?

(possible follow up topics: treatment, pain relief, preparations before surgery and follow-up after surgery)

**Consultation at drop in anesthesia clinic**

How did you experience the consultation at the drop in anesthesia clinic?

(possible follow up topics: involvement, pain relief, preparations before surgery and follow-up after surgery)

**Waiting for surgery**

Were you contacted by the hospital while you waited for surgery?

How did you experience this contact?

**Surgery**

How did you experience the preparations for surgery?

How did you experience the discharge process after surgery?

(possible follow up topics: information, prescriptions, pain therapy, wound treatment, discharge letter)

**Miscellaneous**

What were your main expectations prior to these sequences of treatment?

Were your expectations met?

Are there any other experiences that you would like to tell me about?
