# Supplementary material for: Patient experiences with interventions to reduce surgery cancellations: a qualitative study
Source: BMC Surg. 2013 Aug 8;13:30. doi: 10.1186/1471-2482-13-30 (PMC3750692; doi:10.1186/1471-2482-13-30)
Supplement: Additional file 2 — Additional quotes from patients. [file 1471-2482-13-30-S2.docx]

**Additional quotes from patients illustrating our findings**

**About the importance of being involved in deciding time for surgery**

(P): *Yes, for sure. You need to be able to plan and to inform your employer that you’ll be gone. Knowing when your surgery will be is very important*. P 3

(I): *How did you feel about being given your surgery date when you left your consultation at the out-patient clinic?*

(P): *I thought that was great. Right away you know what the plan is.* P 2

(I): *Were you involved in scheduling your surgery?*

(P): *No. I just had to wait and it took several months*.

(I): *In some places, patients are assigned surgery slots when they have their consultations at the clinic. Would that have been important for you?*

(P): *Yes, that would have been good, because then you’d know the time and could plan around that instead of having to go and wait and not know anything until you get a letter in the mail.* P 12

**About the phone call two days prior to surgery**

(I): *How did you feel about the hospital calling you two days ahead of your surgery?*

(P): *I was a bit surprised. I felt like they were taking care of things, and that they hadn’t forgotten anything.* P 1

(P): *Their calling you before the surgery is a very good thing. It prepares you for what is to come and you can ask questions if there is anything you wonder about.* P 3

**About relating to few clinicians**

(I): *Are there other things you like to share?*

(P): *If possible, it is a very good thing if the same people are there at the different stages. My own surgery was a simple one, but if I were older, or more disoriented than I am, it would have been hard.* P 7
